# Supplementary material for: Single-transistor organic electrochemical neurons
Source: Nat Commun. 2025 May 9;16:4334. doi: 10.1038/s41467-025-59587-4 (PMC12064751; doi:10.1038/s41467-025-59587-4)
Supplement: Supplementary file 1 — Supplementary Information [file 41467_2025_59587_MOESM1_ESM.pdf]

Supplementary Information

Single-transistor organic electrochemical neurons

Junpeng Ji[1], Dace Gao[1], Han-Yan Wu[1], Miao Xiong[1], Nevena Stajkovic[2,3], Claudia Latte Bovio[4,5], Chi-Yuan Yang[1], Francesca Santoro[2,3,4], Deyu Tu[1], Simone Fabiano[1]*

[1]Laboratory of Organic Electronics, Department of Science and Technology, Linköping University, Norrköping, Sweden.

[2]Institute of Biological Information Processing IBI-3 Bioelectronics, Forschungszentrum Jülich, 52428, Jülich, Germany.

[3]Neuroelectronic Interfaces, Faculty of Electrical Engineering and IT, RWTH Aachen, 52074, Aachen, Germany.

[4]Tissue Electronics, Center for Advanced Biomaterials for Healthcare, Istituto Italiano di Tecnologia, 80125, Naples, Italy.

[5]Dipartimento di Ingegneria Chimica, dei Materiali e della Produzione Industriale, Università degli Studi di Napoli Federico II, 80125, Naples, Italy.

E-mail: simone.fabiano@liu.se

Table of Contents

Supplementary Notes

Supplementary Note 1.
Memory effect in BBL-based OECTs......7

Supplementary Note 2.
SPICE simulations of the OECmTs. 11

Supplementary Note 3.
SPICE simulations of the 1T-OECNs 13

Supplementary Note 4.
Neural features demonstrated with the 1T-OECNs......15

Supplementary Note 5.
Implementation of Boolean logic operations using the 1T-OECNs. 28

Supplementary Figures

Supplementary Figure1......4

Supplementary Figure 2......5

Supplementary Figure 3......6

Supplementary Figure 4......8

Supplementary Figure 5......9

Supplementary Figure 6......10

Supplementary Figure 7......11

Supplementary Figure 8......12

Supplementary Figure 9......14

Supplementary Figure 10...... 18

Supplementary Figure 11......22

Supplementary Figure 12......23

Supplementary Figure 13......24

Supplementary Figure 14......25

Supplementary Figure 15......26

Supplementary Figure 16......27

Supplementary Figure 17......29

Supplementary Figure 18......30

Supplementary Figure 19......31

Supplementary Figure 20 32
Supplementary Figure 21 33
Supplementary Figure 22 34
Supplementary Figure 23 35
Supplementary Figure 24 36
Supplementary Figure 25 37

Supplementary Tables

Supplementary Table 1.
Parameters used for different 1T-OECN configurations 19

Supplementary Table 2.
Comparison of various artificial neuron technologies 21

Reference 38

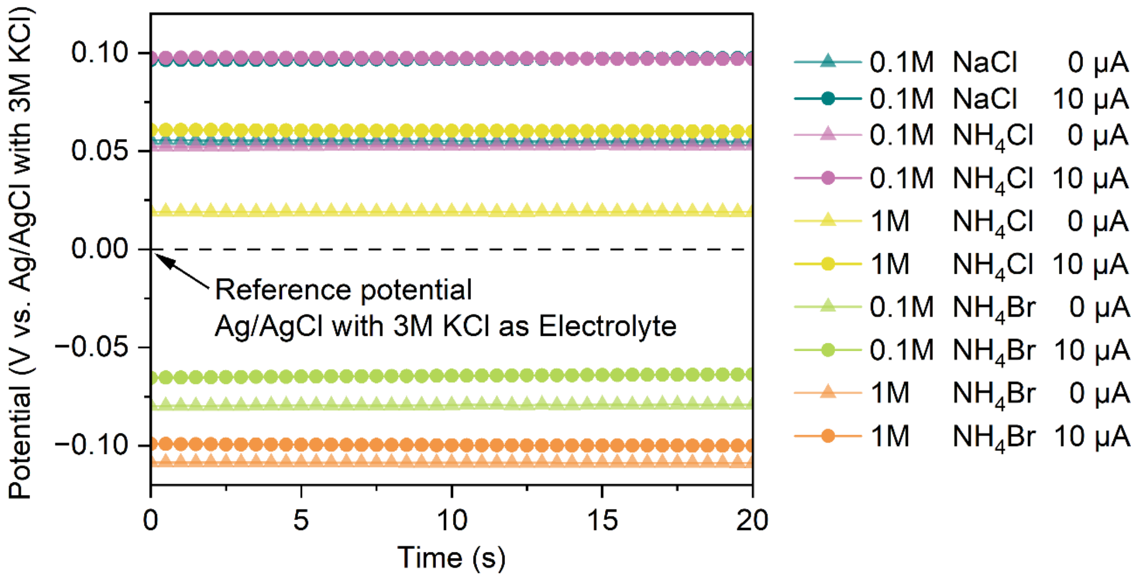

Supplementary Figure 1. Potential calibration of the Ag/AgCl gate electrode in electrolytes containing various concentrations of Cl^- and Br^- ions. Electrolytes with the same ion type and concentration are marked in the figure with the same color. For each electrolyte, the potential difference between the Ag/AgCl electrode and a standard Ag/AgCl electrode in 3M KCl was tested under applied currents of 0 µA (triangle symbols) and 10 µA (circle symbols). When 1 M NH_4Br is used as the electrolyte, the gate electrode exhibits the lowest potential relative to the standard electrode. Under an applied current of 10 µA (maximum gate current observed in this study during normal OECT operation), the potential difference with 1 M NH_4Br is about 0.2 V compared to 0.1 M NaCl or NH_4Cl, and about 0.16 V compared to 1 M NH_4Cl. This trend aligns with the V_p shift observed with different electrolyte concentrations and ion types, as shown in Figure 1c.

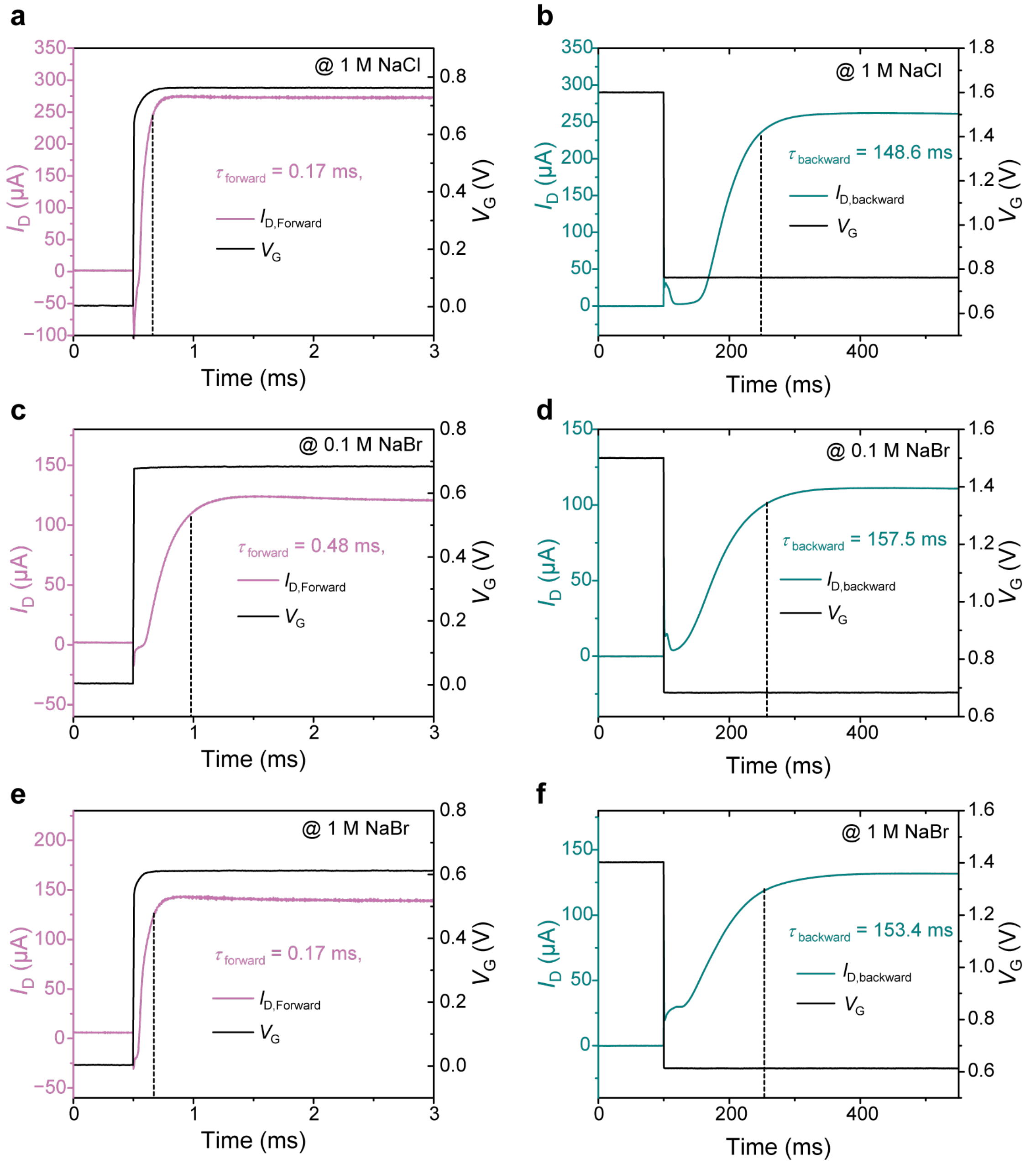

Supplementary Figure 2. Asymmetric switching speed in Na^+-based electrolytes. Turn-on times for OECTs activated from the doped ($\tau_{forward}$) and highly doped ($\tau_{backward}$) states using 1 M NaCl (**a, b**), 0.1 M NaBr (**c, d**), 1 M NaBr (**e, f**). The data for 0.1 M NaCl are presented in Figure 1e. $V_D = 0.7$ V for all measurements above.

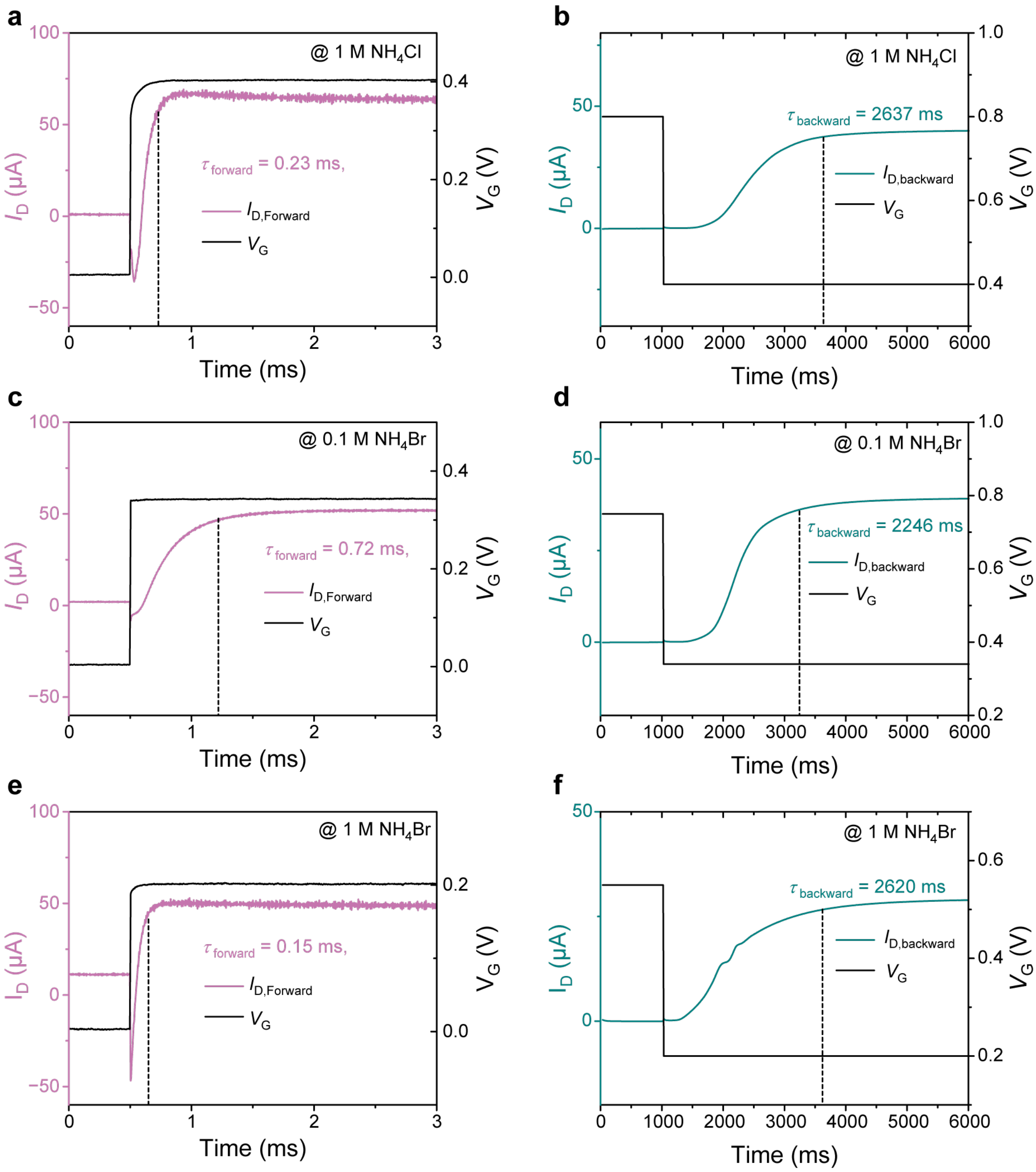

Supplementary Figure 3. Asymmetric switching speed in NH_4^+-based electrolytes. Turn-on times for OECTs activated from the doped ($\tau_{forward}$) and highly doped ($\tau_{backward}$) states using 1 M NH_4Cl (**a, b**), 0.1 M NH_4Br (**c, d**), 1 M NH_4Br (**e, f**). The data for 0.1 M NH_4Cl are presented in Figure 1e. $V_D = 0.3$ V for all measurements above.

Supplementary Note 1. Memory effect in BBL-based OECTs

As shown in Supplementary Figure 4a, V_D is first swept from 0 V in the positive direction, reaching a maximum of 0.6 V, and then swept back to 0 V (sweep 1-2 in Supplementary Figure 4a). When V_G is below 0 V, BBL remains in a normally doped state regardless of changes in V_D, leading to fast switching and minimal delay. In the range of 0.1 V $\leqslant$ V_G $\leqslant$ 0.5 V (as also shown in Figure 1f), BBL enters the highly doped state at low V_D, resulting in slower switching. As V_D increases, BBL transitions back to the doped state, causing a rapid turn-on of the OECT. When V_D is swept back toward 0 V, BBL re-enters the highly doped state, leading to a slow OECT turn-off. Thus, during a full positive V_D sweep cycle, the channel current experiences an initial slow turn-on in the highly doped state region, a rapid turn-on in the doped state region, followed by a gradual turn-off as it returns to the highly doped state region.

Next, V_D is swept from 0 V in the negative direction, reaching -0.6 V, and then swept back to 0 V (sweep 3-4 in Supplementary Figure 4a). When V_G is below 0.2 V, at the beginning of the negative sweep, BBL is in the doped state, resulting in a fast OECT switch-on. As the sweep progresses to more negative bias, BBL transitions to the highly doped state, causing the OECT to remain on but with a slower current change. As the sweep returns to a smaller negative bias, the OECT switches back to the doped state, quickly turning off after reaching a current peak. However, when V_G exceeds 0.2 V, BBL remains in the highly doped state throughout the sweep, and the current stays at a low level for the entire sweep. A similar phenomenon is observed when using 1 M NaCl as the electrolyte (Supplementary Figure 4b), but only when V_G is fixed at 0.7 V, showing much smaller hysteresis compared to 1 M NH_4Br. It is important to note that a V_G of 0.7 V is relatively high and can hinder the stable operation of the OECT if applied continuously. As shown in Supplementary Figure 5b, larger hysteresis can also be observed in OECTs with 1 M NaCl at higher V_G and faster scan rates. However, excessively high V_G makes it unsuitable for use in neuromorphic devices. Supplementary Figure 6 shows that when V_D is open and V_G is turned on from 0 V, there is no delay in the channel current turning on. However, when V_G is maintained at an appropriate level and V_D is turned on from 0 V, a delay in channel current activation is observed, corresponding to the hysteresis in the output curve.

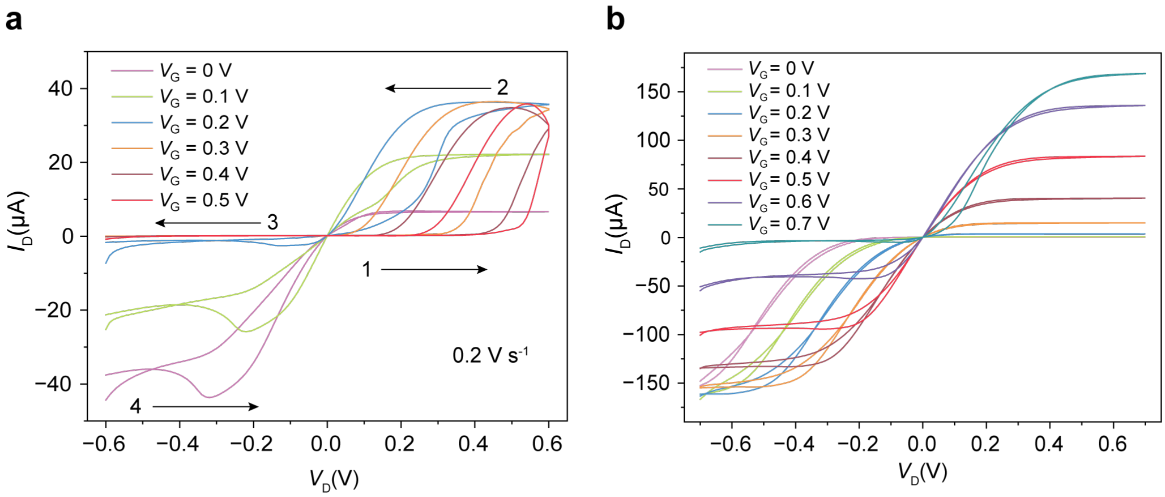

Supplementary Figure 4. a, Output curves of BBL-OECTs showing hysteresis. The numbers and arrows indicate the scanning order and direction of V_D. Measurements were performed with a scan rate of 0.2 V s^{-1} using 1 M NH_4Br as the electrolyte. **b**, Output curve of BBL OECTs using 1 M NaCl as the electrolyte for comparison. When V_D is scanned in the positive direction, only a small hysteresis is observed at V_G = 0.7 V (scan rate of 0.2 V s^{-1}).

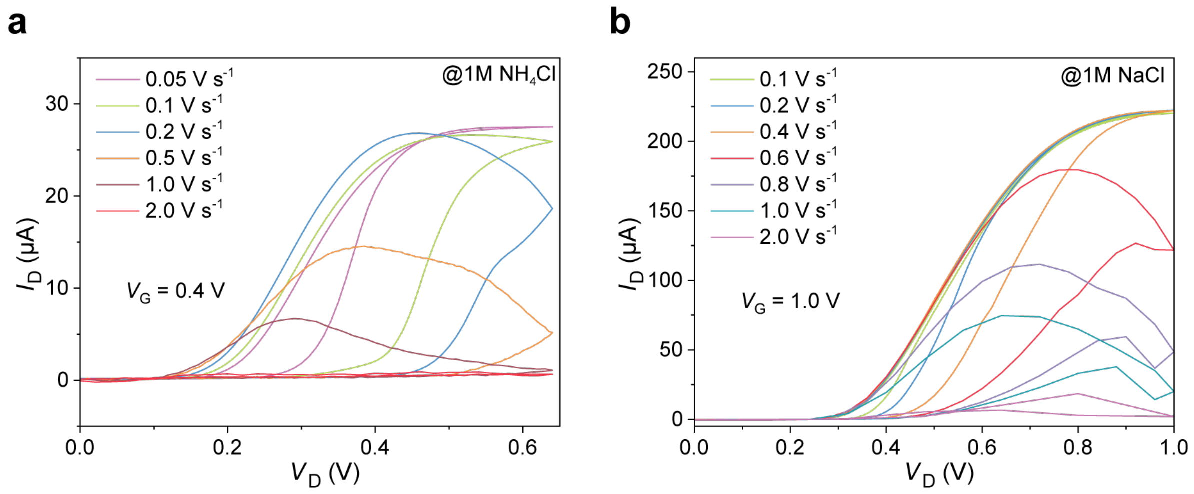

Supplementary Figure 5. Hysteresis behavior of BBL-OECmTs at different scan rates using 1 M NH_4Br (**a**) and 1 M NaCl (**b**). At slower scan rates, the hysteresis is maintained. At faster scan rates (>2.0 V s^{-1}), the hysteresis shrinks, forming a nearly straight line at a low I_D level.

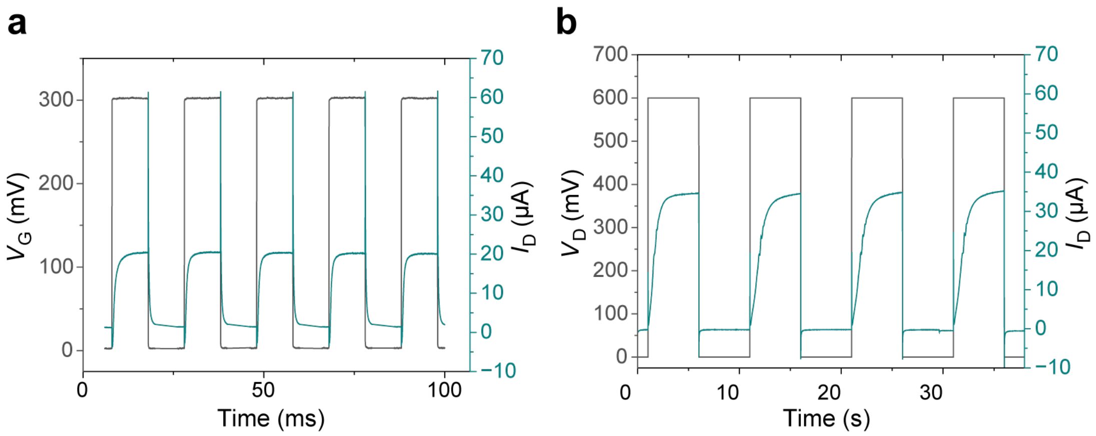

Supplementary Figure 6. Comparison of turn-on speed when activating the channel using gate and drain voltages with 1 M NH_4Br electrolyte. **a**, Evolution of I_D when V_G is switched from 0 V to 0.3 V, while V_D is maintained at 0.3 V. When V_G is turned on, the I_D activation speed is at the millisecond level. **b**, Evolution of I_D when V_D is switched from 0 V to 0.6 V, while V_G is maintained at 0.4 V. When V_D is turned on, the I_D activation speed is at the second level.

Supplementary Note 2. SPICE simulations of the OECmTs

With NH_4^+-based electrolytes, the output characteristics of BBL-OECTs exhibit significant hysteresis. To simulate this behavior, we developed a Schmitt trigger module using both p-type and n-type OECTs, as reported in our previous work[1]. The Schmitt trigger is then coupled to the drain of a Gaussian (antiambipolar) n-OECT[2], as shown in Supplementary Figure 7a. The non-inverting Schmitter trigger is implemented using eight regular accumulation-mode OECTs (Supplementary Figure 7b). By introducing the Schmitt trigger, we successfully simulate the hysteresis observed in OECTs with NH_4^+-based electrolytes (Supplementary Figure 7c). The sweep rate of the applied V_D is 0.2 V s^{-1}, matching that of the experimental measurements. The simulated hysteresis shows good agreement with the experimental results. However, the transition appears steeper in the simulation, which can be attributed to the faster switching of the accumulation-mode OECTs in the Schmitt trigger module.

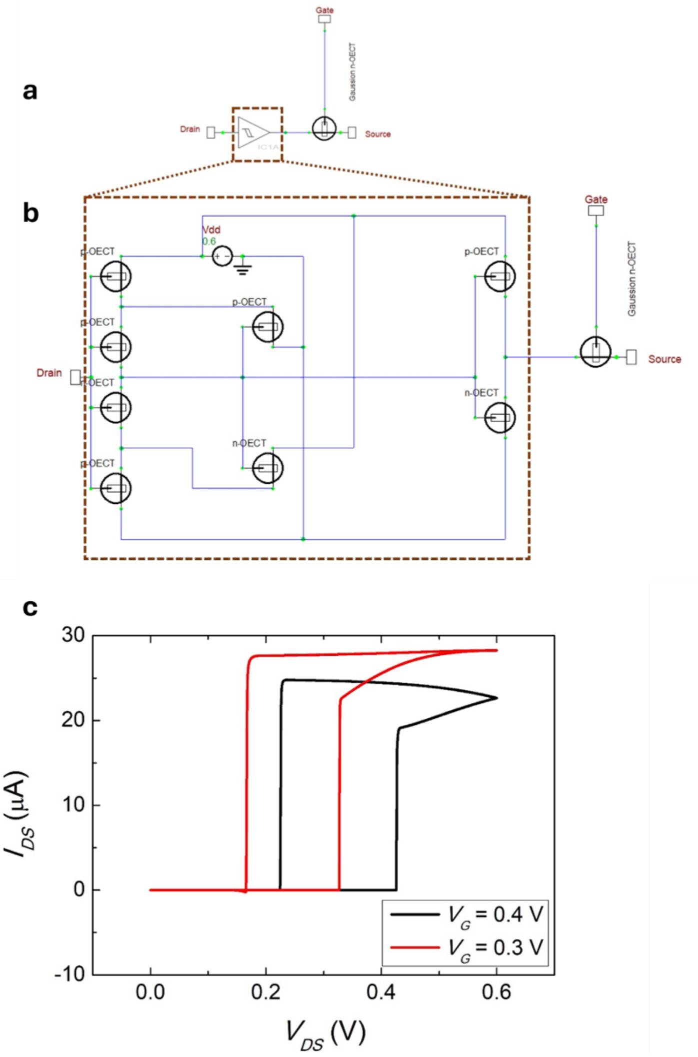

Supplementary Figure 7. a, Equivalent circuit schematic used to simulate BBL-OECTs with NH_4^+-based electrolytes. **b**, Schematic of a Schmitt trigger module implemented using accumulation-mode OECTs. **c**, Simulated output characteristics of BBL-OECTs with large hysteresis.

As discussed in the main text, hysteresis can also be observed in BBL OECTs with NaCl as the electrolyte. However, it occurs at high voltages and is less pronounced. By adjusting the parameters in the Gaussian OECT model (Supplementary Figure 7a,b) to match the characteristics of BBL-OECTs with NaCl, we can simulate the less significant hysteresis, as shown in Supplementary Figure 8. However, due to the small hysteresis, this model does not exhibit spiking behaviors in the 1T-OECN simulation.

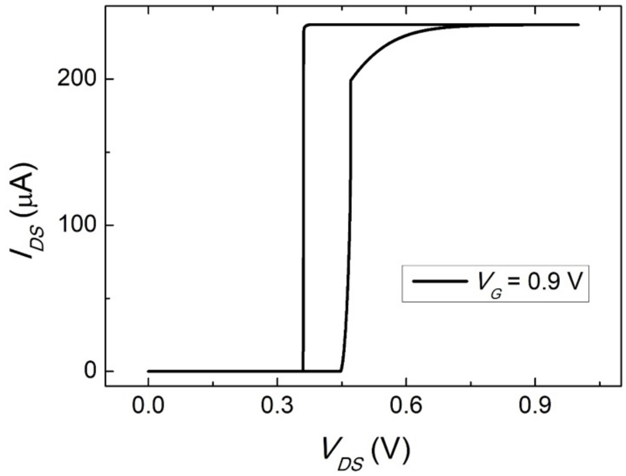

Supplementary Figure 8. Simulated output characteristics of BBL-OECTs with NaCl (small hysteresis).

Supplementary Note 3. SPICE simulations of the 1T-OECNs

Given the presence of the Schmitt trigger in the equivalent circuit (Supplementary Figure 7a), the neural spiking features of 1T-OECNs can be interpreted as the outcome of a Schmitt trigger oscillator. Supplementary Figure 9a presents the equivalent circuit schematic of a 1T- OECN, where a resistor ($R_{G,leak}$) and a capacitor (C_{GS}) are used to regulate the spiking frequency. The resistor $R_{G,leak}$ represents the leakage resistance through the gate of the accumulation-mode OECT, while the capacitor C_{GS} accounts for the gate-to-source capacitance. Three exemplary neural spiking patterns (Tonic, Class 2, and Phasic) are shown in Supplementary Figure 9.

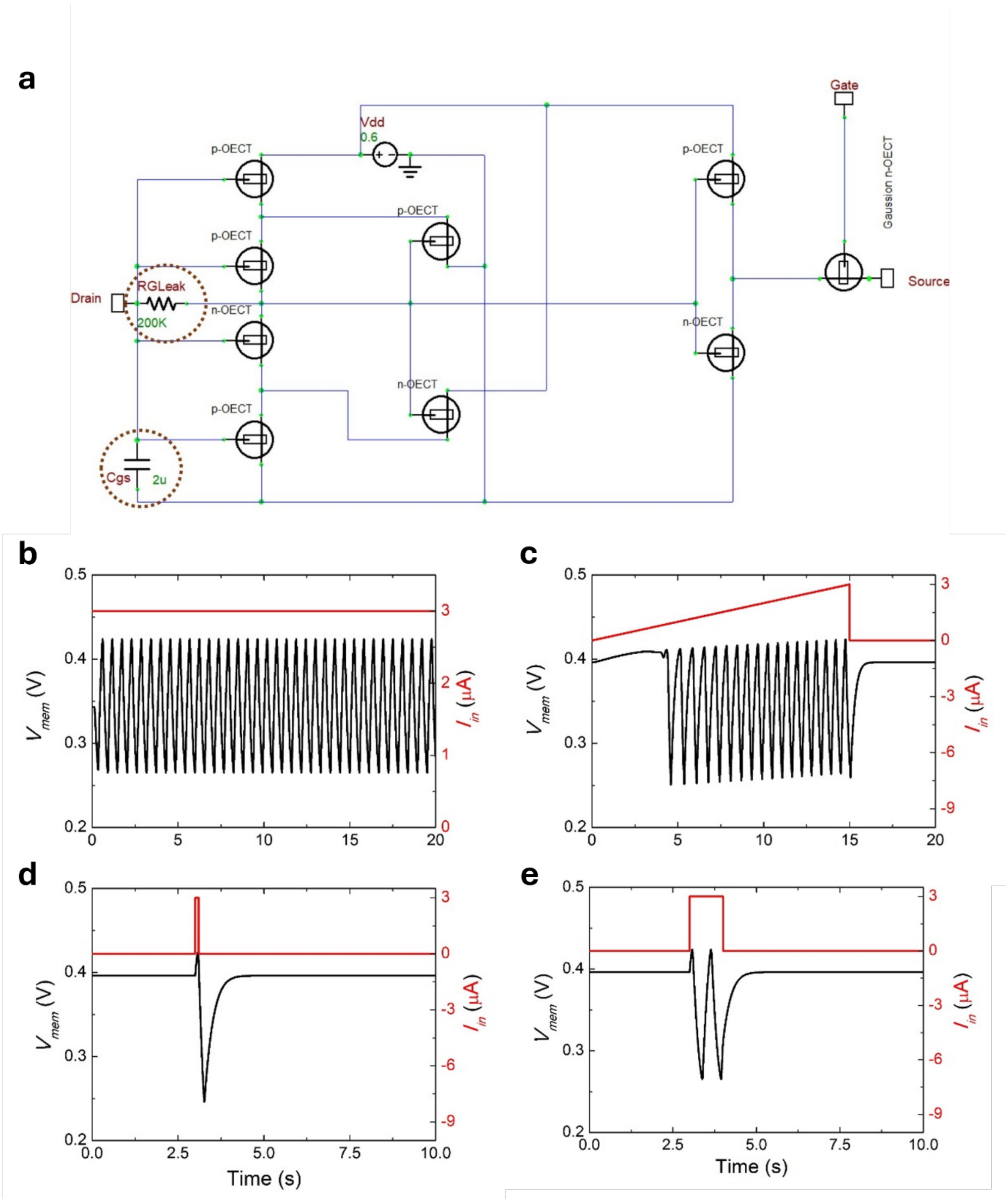

Supplementary Figure 9. **a**, Equivalent circuit of a 1T-OECN. **b**, Simulated tonic spiking under a constant current input. **c**, Simulated class 2 spiking behavior. **d,e**, Simulated phasic spiking responses.

Supplementary Note 4. Neural features demonstrated with the 1T-OECNs

We explored a range of neural features experimentally demonstrated in the 1T-OECN devices, drawing parallels with their biological counterparts. By examining key features such as spiking behavior, adaptation, and resonance, we aim to highlight the functionality in both artificial and biological neural systems.

The following characteristics have been reported in various artificial neuron models (such as the Hodgkin-Huxley model, the Izhikevich model, etc.)[3–5]:

1) Class 1 (C1) (Figure 3b, left): Class 1 spiking neurons are characterized by a linear response to input currents, allowing for a gradual and continuous increase in firing rates. Unlike Class 2 spiking neurons, which show a distinct threshold for spiking, Class 1 spiking neurons do not have a sharp threshold. Instead, they can exhibit a continuous transition from resting potential to spiking. This type of spiking behavior is crucial for encoding information and processing signals in various neural systems[6,7].
2) Class 2 (C2) (Figure 3b, middle): Class 2 neurons exhibit a clear threshold for spiking. When the input current reaches a certain level (the threshold), the neuron rapidly generates action potentials (spikes). Below this threshold, the neuron may remain quiescent or produce very few spikes. The relationship between the input current and the firing rate in Class 2 neurons is nonlinear. As the input current increases beyond the threshold, the firing frequency can increase rapidly, often in a dynamic and sometimes irregular manner. This behavior is essential for processing temporal information, generating rhythmic activity, and enabling dynamic responses to stimuli in various neural systems[7].
3) Phasic spiking (PS) (Figure 3b, right): Also known as class 3 spiking. Even with very high current injections, Class 3 neurons typically exhibit only a single action potential or a few spikes[8]. For Class 3 excitatory neurons, a single action potential is usually generated in response to current pulses of varying intensities, and only under extremely strong current injections do they produce three to five spikes[9].
4) Biophysically meaningful (BM) (Figure 3d): The characteristic of artificial neurons influencing their working state through ion response reflects biophysically meaningful properties. Ion response in neurons refers to the way a neuron reacts to the movement of ions across its membrane, which is crucial for generating electrical signals such as action potentials. This process involves various types of ion channels that regulate the flow of ions, primarily sodium (Na^{+}), potassium (K^{+}), calcium (Ca^{2+}), and chloride (Cl^{-}), in and out of the neuron. Changes in the ion environment (concentration and type) have a decisive impact on the spiking state of neurons[10].
5) Tonic spiking (TS) (Supplementary Figure 11): Neurons can continuously fire action potentials at an almost constant frequency under continuous or long-term external stimulation[5]. This pattern of electrical activity is characterized by continuous, stable firing of action potentials.
6) Tonic bursting (TB) (Figure 3d, right): Tonic bursting refers to a type of neural firing pattern where a neuron exhibits periods of rapid action potential bursts (groups of spikes) followed

by quiescent periods, all while maintaining a relatively constant external stimulus. Unlike tonic spiking, where the neuron fires at a steady, regular rate, tonic bursting involves clusters of action potentials separated by intervals of little or no activity[11].

7) Mixed-mode (M) (Supplementary Figure 12c): Mixed-mode refers to neurons showing multiple different firing patterns within the same time period. This behavior is common in certain types of neurons, especially when they are affected by complex electrophysiological inputs or intrinsic conditions[4]. In mixed mode, neurons may exhibit subthreshold oscillations (i.e., changes in voltage that do not reach the threshold to trigger an action potential) while occasionally firing suprathreshold spikes. This suggests that tiny oscillations in neuron's electrical activity in low-energy states coexist with action potentials in high-energy states[12].
8) Adaptation (Ad.) (Supplementary Figure 12d): Spike frequency adaptation refers to the phenomenon where a neuron's firing rate decreases in response to a constant or sustained stimulus over time. This adaptation allows neurons to respond to changes in input without becoming overly excited or fatigued[13].
9) Chaotic spiking (C) (Supplementary Figure 13a): Chaotic spiking (also known as Random firing) of neurons refers to neurons firing action potentials in an irregular manner. Unlike regular discharge patterns, random discharges do not have fixed time intervals or frequencies but present a random time distribution. This firing behavior can be observed in certain areas of the brain, particularly in neural networks related to perception, decision-making, or learning[20,21].
10) Integration (I) (Supplementary Figure 14a): Integration refers to the ability of neurons to process and combine multiple synaptic inputs to determine whether to generate an action potential. This specific type of integration involves the simultaneous arrival of excitatory inputs at the same time, which can produce a stronger overall signal. When two or more synaptic inputs arrive at the postsynaptic neuron nearly simultaneously, the combined effect can lead to depolarization sufficient enough to reach the threshold for firing an action potential[2,4].
11) Refractory period (RP) (Supplementary Figure 14b): Refractoriness in neurons refers to the period following an action potential during which the neuron is less excitable and has a reduced ability to fire additional action potentials. The refractory periods effectively limit the maximum frequency at which a neuron can fire. Refractoriness ensures that neurons can respond to stimuli in a controlled manner, preventing excessive firing and allowing for the temporal coding of signals. It also contributes to the directional propagation of action potentials along axons, ensuring that signals travel effectively from one part of the nervous system to another[17,18].
12) Resonance (R) (Supplementary Figure 14c): Resonance refers to the phenomenon where a neuron exhibits a heightened response to specific input frequencies. This property enables neurons to preferentially respond to certain oscillatory signals in their environment, which is crucial for various neural functions, like sensory processing and signal integration[2,19].
13) Accommodation (A) (Supplementary Figure 14d): Neurons are likely to fire action potentials in response to rapid changes in input rather than to steady or slowly changing inputs. This behavior is advantageous in biological systems for detecting and responding

to important stimuli that require immediate attention, such as sudden changes in sensory input[20,21].

14) Stochastic resonance (SR) (Figure 3c): In a neuron, weak stimuli may not produce a strong enough response to trigger an action potential. However, when random noise (such as thermal or synaptic noise) is present, it can interact with the weak signal, effectively pushing the membrane potential above the threshold for firing[2,22].

The following neuronal characteristics reflect the higher-order complexity of neurons[14]:

15) Refractory period plasticity (Supplementary Figure 12a): Refractory period is an inherent characteristic of excitable neuronal elements, introducing unavoidable time delays before re-excitation. In certain neurons, the absolute refractory period (ARP) has been shown to exhibit rapid and significant plasticity, depending on the firing activity of their connected neurons, which is expected to influence network dynamics[23]. In 1T-OECNs, the refractory period can be extended, demonstrating the plasticity of the refractory period.
16) Damped spiking (Supplementary Figure 13b): A neuron might fire rapidly at first, then gradually slow down and stop spiking as the stimulus continues or inhibitory feedback increases[9,14]. Damped spiking may occur in neurons that adapt to continuous or prolonged stimulation.
17) Beyond-threshold damped oscillations (Supplementary Figure 13c,d): Damped oscillations typically occur below threshold[5], but recent findings indicate they can also persist above threshold, influencing nonlinear computations in dendritic neurons[1424].

To achieve these features, we used the OECT architectures reported in Supplementary Figure 10 and applied the electrical signals detailed in Supplementary Table 1.

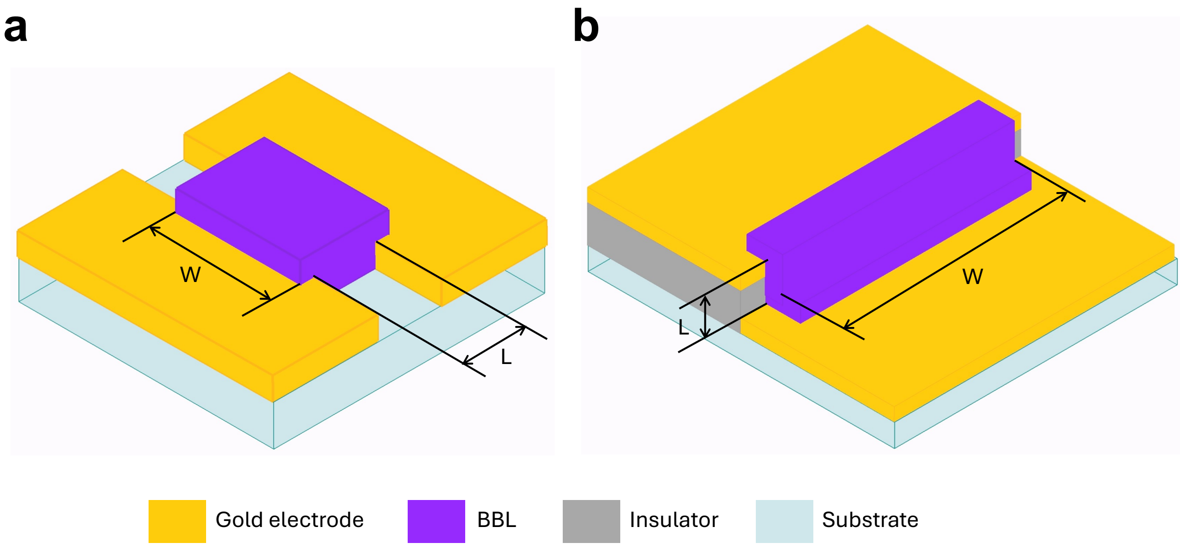

Supplementary Figure 10. Two 1T-OECN architectures were used in this work: planar (**a**) and vertical (**b**). The channel length (L) and width (W) are labeled for each structure.

Supplementary Table 1. Parameters used for different 1T-OECN configurations.

	Figures	Feature	Structure	Electrolyte	V_G (V)	V_S (V)
1	Figure 3b	Class1 spiking	Planar W = 200 μm, L = 6 μm	1M NH_4Br	0.45	0
2		Class2 spiking	Planar W = 200 μm, L = 6 μm	1M NH_4Br	0.35	-0.2
3		Class3 spiking (Phasic spiking)	Planar W = 200 μm, L = 6 μm	1M NH_4Br	0.35	0
4	Figure 3c	Stochastic resonance	Planar W = 200 μm, L = 6 μm	1M NH_4Br	0.3	0
5	Figure 3d (left)	Biophysically meaningful (Ion response)	Planar W = 200 μm, L = 6 μm	0.1 M NH_4Br	0.45	0
	Figure 3d (middle)		Planar W = 200 μm, L = 6 μm	0.1 M NH_4Cl	0.6	0
6	Figure 3d (right)	Tonic bursting	Planar W = 200 μm, L = 6 μm	0.1 M NH_4Cl	0.6	0
7	Figure 4f	Sensor module integration	Vertical W = 300 μm, L < 1 μm	1M NH_4Br	0.5	0
8	Supplementary Figure 11	Tonic spiking	Planar W = 20 μm, L = 10 μm	1M NH_4Br	0.3	0
9	Supplementary Figure 12a	Refractory period plasticity	Vertical W = 800 μm, L < 1 μm	1M NH_4Br	0.37	0
10	Supplementary Figure 12b	Class3 spiking (Phasic spiking)	Planar W = 200 μm, L = 6 μm	1M NH_4Br	0.38	-0.1
11	Supplementary Figure 13c	Mixed mode	Planar W = 200 μm, L = 6 μm	1M NH_4Br	0.48	0
12	Supplementary Figure 12d	Adaptation	Vertical W = 300 μm, L < 1 μm	1M NH_4Br	0.35	0
13	Supplementary Figure 13a	Chaotic spiking (Random firing)	Planar W = 400 μm, L = 6 μm	1M NH_4Br	0.33	0
14	Supplementary Figure 13b	Damped spiking	Vertical W = 800 μm,	1M NH_4Br	0.45	-0.01

			L < 1 μm			
15	Supplementary Figure 13c	Beyond-threshold damped oscillations	Planar W = 200 μm, L = 6 μm	0.1 M NH_4Cl	0.6	0
16	Supplementary Figure 14a	Integration	Planar W = 200 μm, L = 6 μm	1M NH_4Br	0.35	-0.05
17	Supplementary Figure 14b	Refractoriness	Planar W = 200 μm, L = 6 μm	1M NH_4Br	0.35	-0.05
18	Supplementary Figure 14c	Resonance	Planar W = 200 μm, L = 6 μm	1M NH_4Br	0.35	-0.05
19	Supplementary Figure 14d	Accommodation	Planar W = 200 μm, L = 6 μm	1M NH_4Br	0.35	-0.1
20	Supplementary Figure 15	Regulation of current stimulation	Planar W = 400 μm, L = 6 μm	1M NH_4Br	0.47	0
21	Supplementary Figure 16a	Voltage regulation	Planar W = 400 μm, L = 6 μm	1M NH_4Br	0.35 to 0.57	0
	Supplementary Figure 16b		Planar W = 400 μm, L = 6 μm	1M NH_4Br	0.47	-0.08 to 0.1

Supplementary Table 2. Comparison of various artificial neuron technologies.

Material / technology	Neuron model	Circuit elements (excluding power sources)	Neuron features	Ion-based modulation	Spike swing	Footprint (μm^2)	Ref.
Silicon	LIF	>10	4	No	300 mV	993	25
Silicon	FitzHugh–Nagumo	>10	11 (modeling)	No	2500 mV	/	26
Silicon	UCN LIF	>10	12	No	1000-4000 mV	/	27
Silicon	Mihalas–Niebur	>10	15 (simulation)	No	1000 mV	2475	28
Mott memristors	Simplified Hodgkin-Huxley	8	5	No	500 mV	/	29
Mott memristors	Adaptive LIF	9	4	No	2000 mV	41.3 - 53.4	30
Mott memristors	Hodgkin-Huxley	6	14 (Without changing circuit)	No	1000 mV	/	4
Mott memristors	Multi-order complexity	3	15	No	2 mA	< 0.01	14
2D materials	Hodgkin-Huxley	6	8	No	500 mV	/	31
2D materials	LIF	1	1	No	2 μA	/	32
2D materials	LIF	1	2	No	10 mV	/	33
OECTs	LIF	7	4	Yes	600 mV	1.2×10^9	1
OECTs	LIF	7	4	Yes	700 mV	3.7×10^7	34
OECTs	Hodgkin-Huxley	6	15	Yes	100 - 200 mV	$> 2 \times 10^8$	2
OECTs	S-NDR	6	6	Yes	100 μA	/	35
**Single OECT**	**Multi-order complexity**	**1**	**17**	**Yes**	**200 - 800 mV**	**177-1600**	**This work**

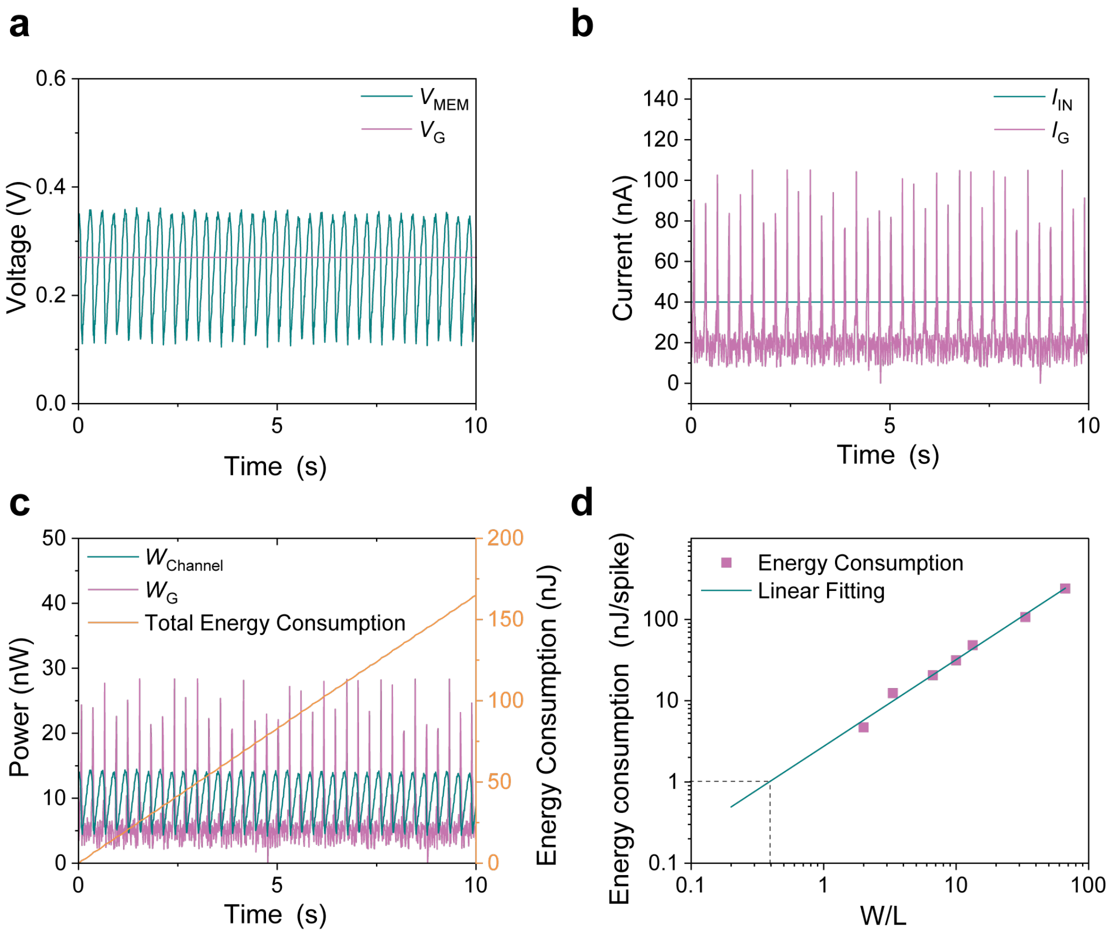

Supplementary Figure 11. Power consumption during tonic spiking. **a**, Tonic spiking of V_{MEM} in 1T-OECN, along with the applied V_G. **b**, Constant input current (I_{IN} = 40 nA) and variation of gate current (I_G) over time during tonic spiking. **c**, Real-time power consumption of both the transistor channel ($W_{Channel}$) and the gate terminal (W_G), along with the calculated total energy consumption. Over 35 cycles of tonic spiking in 10 s, a total of 164.3 nJ of energy is consumed, averaging 4.69 nJ per spike. The average power consumption in tonic spiking mode is 16.43 nW. The measurements were conducted using a 1T-OECN with a channel width of 10 μm and a channel length of 20 μm. **d**, Relationship between energy consumption per spike and the W/L ratio under threshold current stimulation that induces tonic spiking. The solid line represents the linear fitting result. Two channel lengths were used: 6 μm and 10 μm.

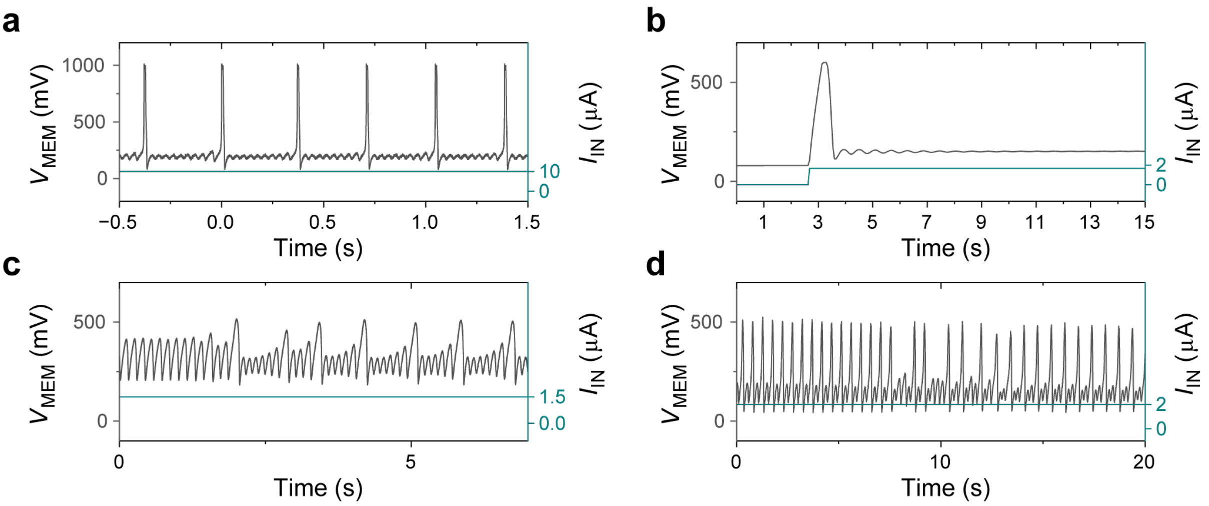

Supplementary Figure 12. Experimental demonstration of various firing features generated by 1T-OECNs: (**a**) refractory period plasticity, (**b**) phasic spiking, and (**c**) mixed mode. **d**, Spike frequency adaptation. Supplementary Table 1 describes the conditions for implementing each firing mode.

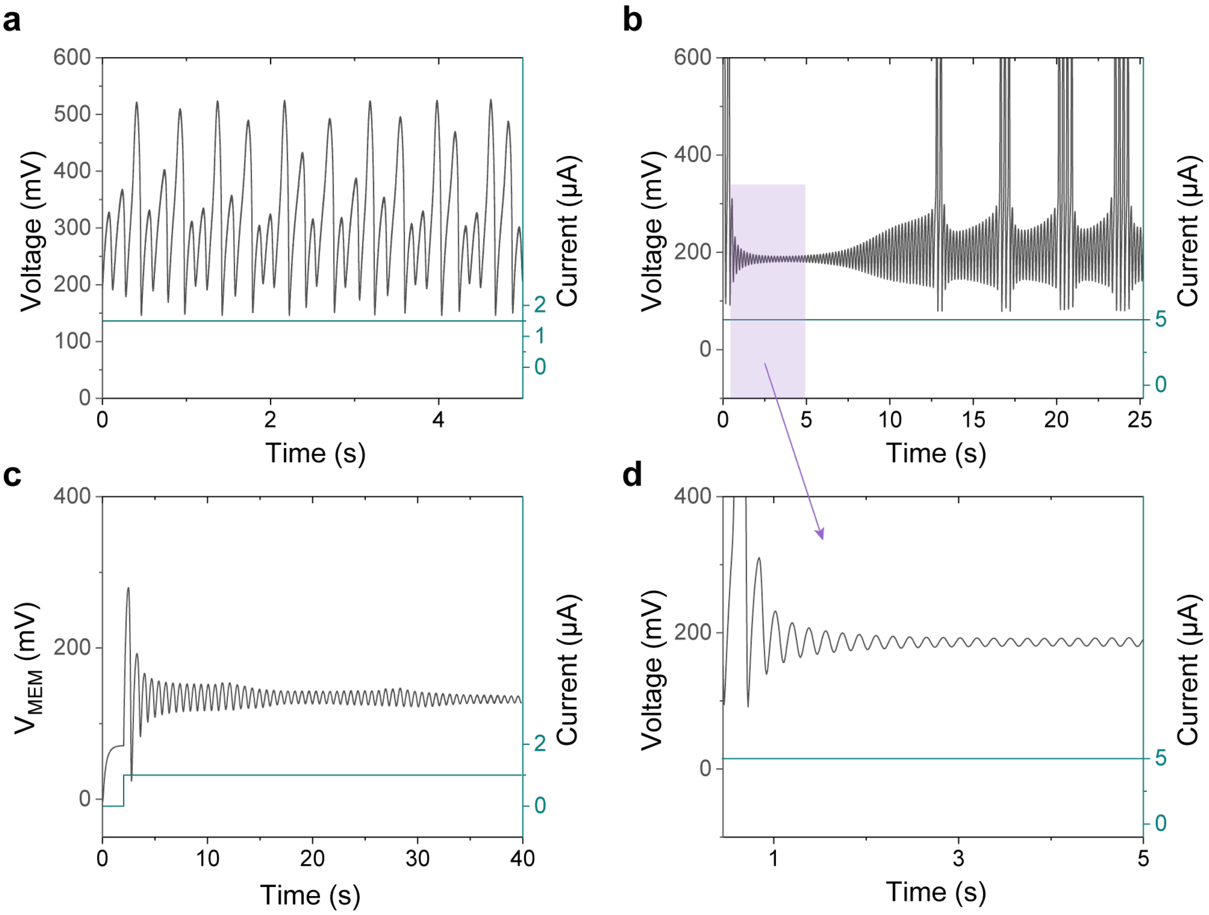

Supplementary Figure 13. High-order complexity spiking features of 1T-OECNs: (**a**) chaotic spiking (random firing) , (**b**) damped spiking, (**c**,**d**) beyond-threshold damped oscillations.

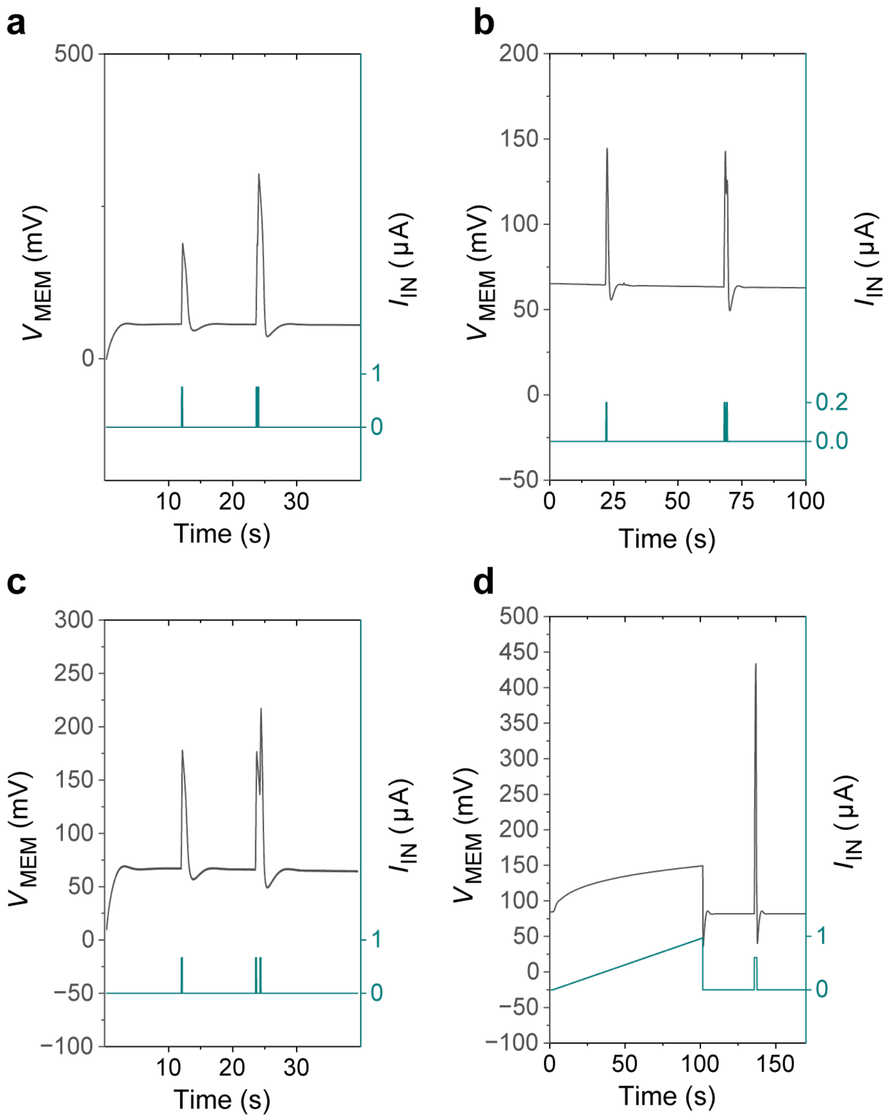

Supplementary Figure 14. Spiking modes of 1T-OECNs under non-continuous independent current stimulation: (**a**) integration, (**b**) refractoriness, (**c**) resonance, and (**d**) accommodation. Supplementary Table 1 describes the conditions for implementing each firing mode.

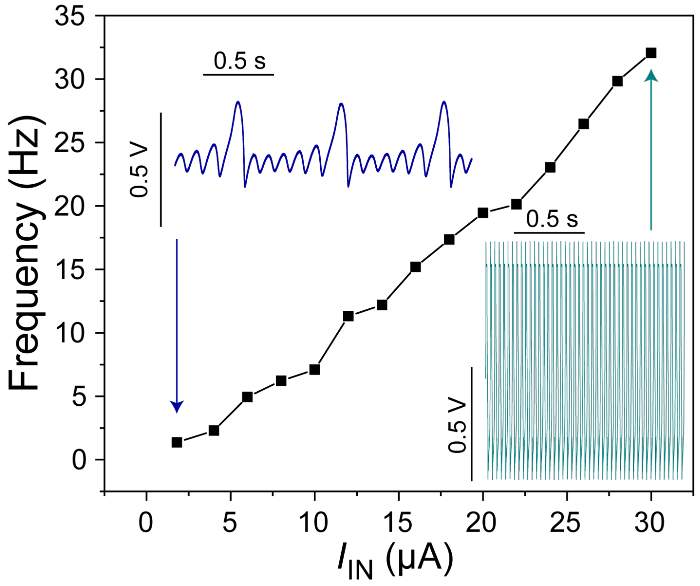

Supplementary Figure 15. Output spiking frequency as a function of the applied input current to the 1T-OECN. The insets illustrate the spiking waveform at minimum (left) and maximum (right) spiking frequency.

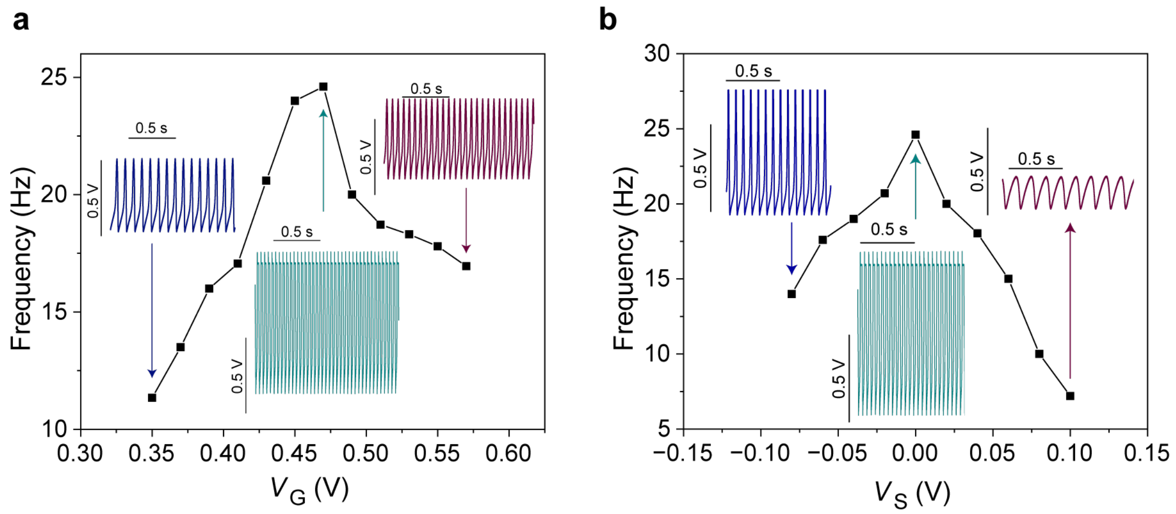

Supplementary Figure 16. Voltage regulation of 1T-OECN output peak frequency with a fixed current stimulation of 25 μA. **a**, Relationship between applied V_G and 1T-OECN output spike frequency. As V_G increases, the output frequency first increases to a peak value and then decreases. The insets illustrate the spiking waveform at minimum V_G (left), peak V_G (medium), and maximum V_G (right). **b**, Relationship between applied V_S and 1T-OECN output spike frequency. As V_S increases, the output frequency first increases to a peak value and then decreases. The insets illustrate the spiking waveform at minimum V_S (left), V_S with the fastest spiking frequency (medium), and maximum V_S (right).

Supplementary Note 5. Implementation of Boolean logic operations using the 1T-OECNs

As discussed in the main text, 1T-OECN can perform six types of Boolean logic operations—AND, OR, XOR, NAND, NOR, and XNOR—without altering the wiring. These operations are realized simply by using different input patterns. Supplementary Figure 17 shows the truth table for the six Boolean logic calculations with two inputs. Below, we describe the specific implementation of each logical operation:

1) AND and OR operations (Figure 3f): Two input variables, Input X and Input Z, are used, while Input Y is kept constant (0 V for AND and -0.1 V for OR). Input X is defined as the "false" logic state (0) at 0.26 V and the "true" logic state (1) at 0.4 V. Similarly, Input Z is defined as the "false" logic state (0) at 0.3 μA and the "true" logic state (1) at 0.8 μA. For the AND operation, 1T-OECN starts to output spikes only when both Input X and Input Z are in the (1) state. For the OR operation, 1T-OECN starts to output spikes when at least one of the two inputs is in the (1) state.

2) XOR and NAND operations (Figures 3g). Input Y is always fixed at 0 V, Input Z is always fixed at 0.5 μA, and Input X_1 and Input X_2 are used as the two input variables. It should be noted that Input X_1 and Input X_2 are combined into Input X, which acts as the V_G of 1T-OECN to affect its spiking state. In the XOR operation, Input X_1 and Input X_2 are defined as the "false" logic state (0) at 0.1 V and the "true" logic state (1) at 0.35 V. 1T-OECN outputs spikes only when the states of X_1 and X_2 are inconsistent. In the NAND operation, Input X_1 and Input X_2 are defined as the "false" logic state (0) at 0.2 V and the "true" logic state (1) at 0.35 V. 1T-OECN does not output spikes only when both X_1 and X_2 are in the 1 state.

3) NOR and XNOR operations (Figures 3h). For both operations, Input X and Input Y are used as the two input variables, while Input Z is kept constant at 0.3 μA. In the NOR operation, Input X is defined as the "false" logic state (0) at 0.3 V and the "true" logic state (1) at 0.6 V, and Input Y is defined as the "false" logic state (0) at 0 V and the "true" logic state (1) at 0.05 V, 1T-OECN outputs spikes only when Input X and Input Y are both 0. In the XNOR operation, Input X is defined as the "false" logic state (0) at 0.3 V and the "true" logic state (1) at 0.6 V, and Input Y is defined as the "false" logic state (0) at 0 V and the "true" logic state (1) at 0.15 V, 1T-OECN outputs spikes only when Input X and Input Y are consistent.

		Mode (0,0)	Mode (1,0)	Mode (0,1)	Mode (1,1)
Inputs	Input 1	0	1	0	1
	Input 2	0	0	1	1
Outputs	AND	0	0	0	1
	OR	0	1	1	1
	XOR	0	1	1	0
	NAND	1	1	1	0
	NOR	1	0	0	0
	XNOR	1	0	0	1

Supplementary Figure 17. Truth table for AND, OR, XOR, NAND, NOR, and XNOR operations with two inputs.

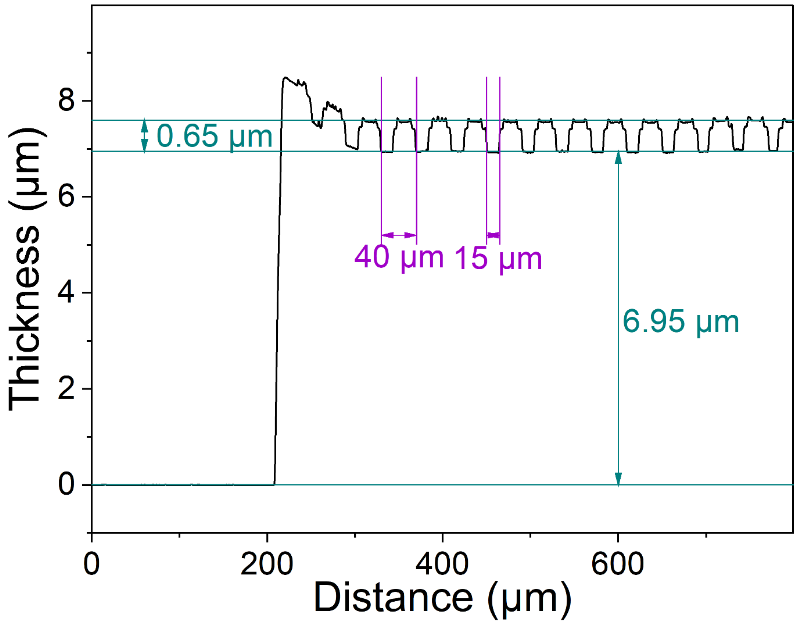

Supplementary Figure 18. Contact profilometer measurement, with the test range spanning from the edge of the array to the 14th 1T-OECN device. The channel diameter of the 1T-OECN device is 15 μm, with a spacing of 40 μm between the center positions of two adjacent 1T-OECNs. The channel depth (length) of the 1T-OECN is 0.65 μm, and the substrate thickness is 6.95 μm.

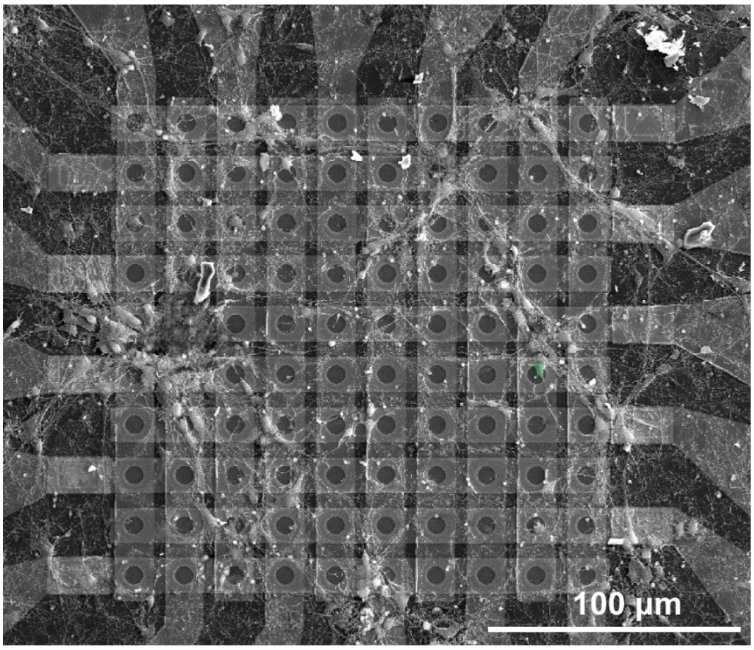

Supplementary Figure 19. Scanning electron micrograph of rat primary cortical neurons on a high-density neuron array after 4 days in vitro. The magnification is 500×.

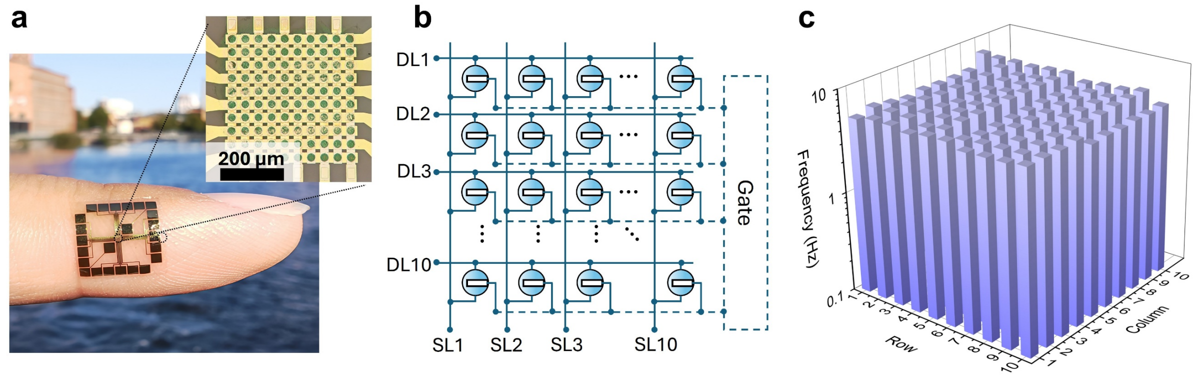

Supplementary Figure 20. a, Photographs of a flexible 10 × 10 1T-OECN array. The structure and density of the unit device are the same as those reported in Figures 4b,c. **b**, Circuit diagram of a 10 × 10 1T-OECN array (DL = drain line, SL = source line). Dashed lines indicate connections through electrolytes rather than physical wiring. **c**, Spiking frequency distribution across the 10 × 10 1T-OECN array. Each device was stimulated by a 5 μA current input with V_G = 0.35 V.

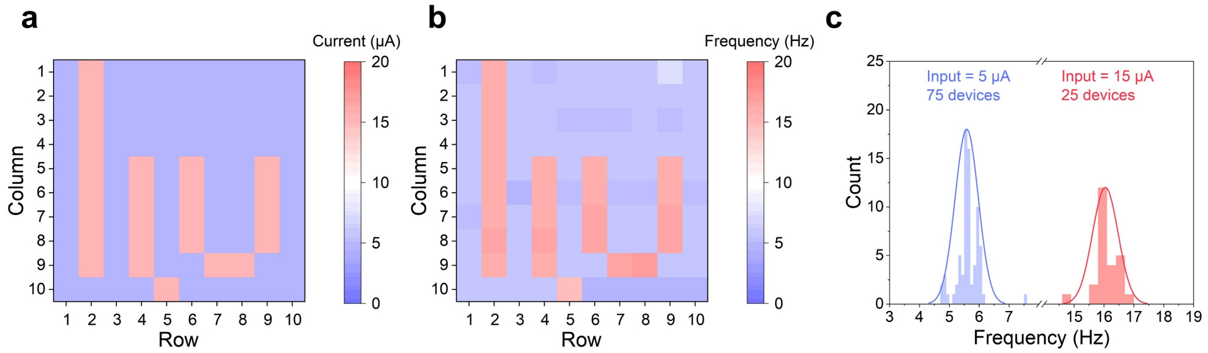

Supplementary Figure 21. **a**, Input current mapping: the 10 × 10 1T-OECN array was programmed to display the Linköping University logo 'lı.u' by applying a 15 μA input current to devices forming the text, while background devices received 5 μA. **b**, Spiking frequency measurement: the input current mapping from (**a**) was applied to all devices. The output peak frequency distribution reproduces the 'lı.u' pattern of the input current. **c**, Oscillation frequency distribution histograms for the 100 1T-OECNs in (**b**): left (low-frequency) region refers to 75 background devices stimulated with a 5 μA, while the right (high-frequency) region refers to 25 devices displaying the 'lı.u' text with a 15 μA current stimulation. Both stimulation levels showed a normal distribution.

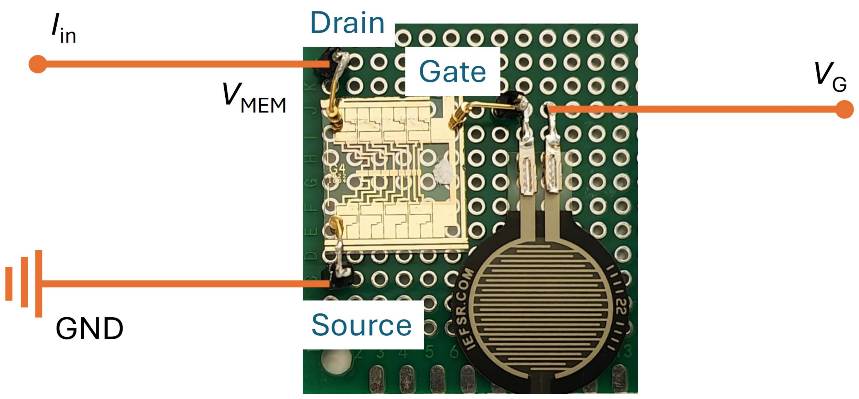

Supplementary Figure 22. Photograph of the artificial afferent nerve chip, composed of one OECT and one touch sensor.

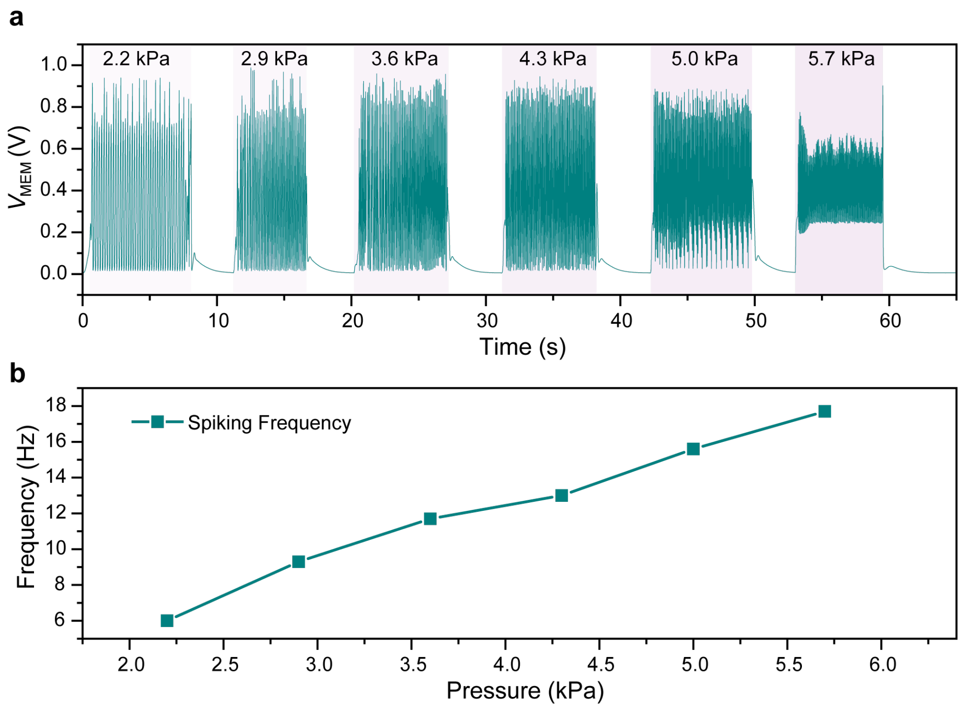

Supplementary Figure 23. a, Spiking voltage output during subsequent pressing-releasing cycles. Different pressures were applied during the tests. **b**, Spiking frequency as a function of applied pressure.

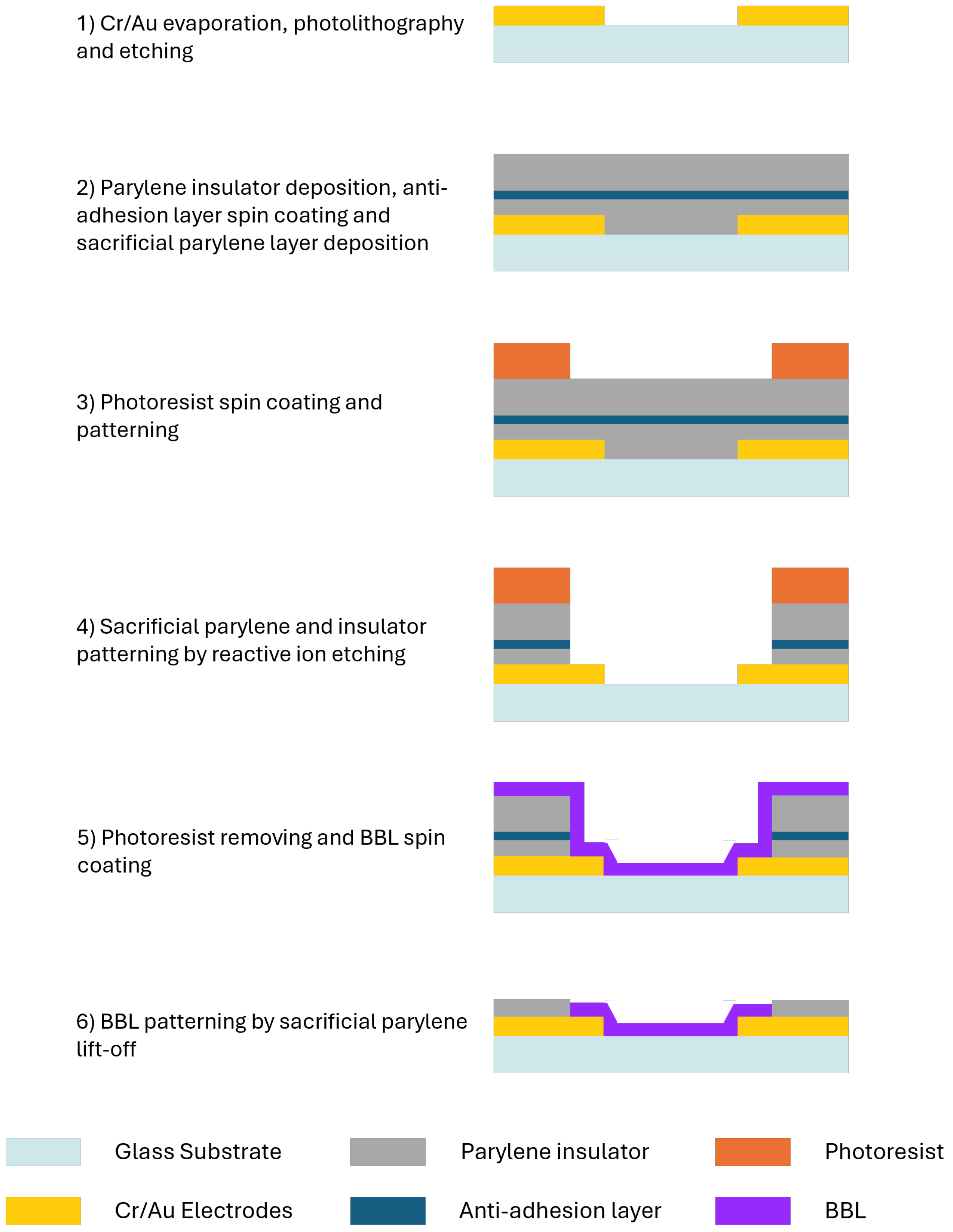

Supplementary Figure 24. Schematic of the fabrication process for a planar 1T-OECN.

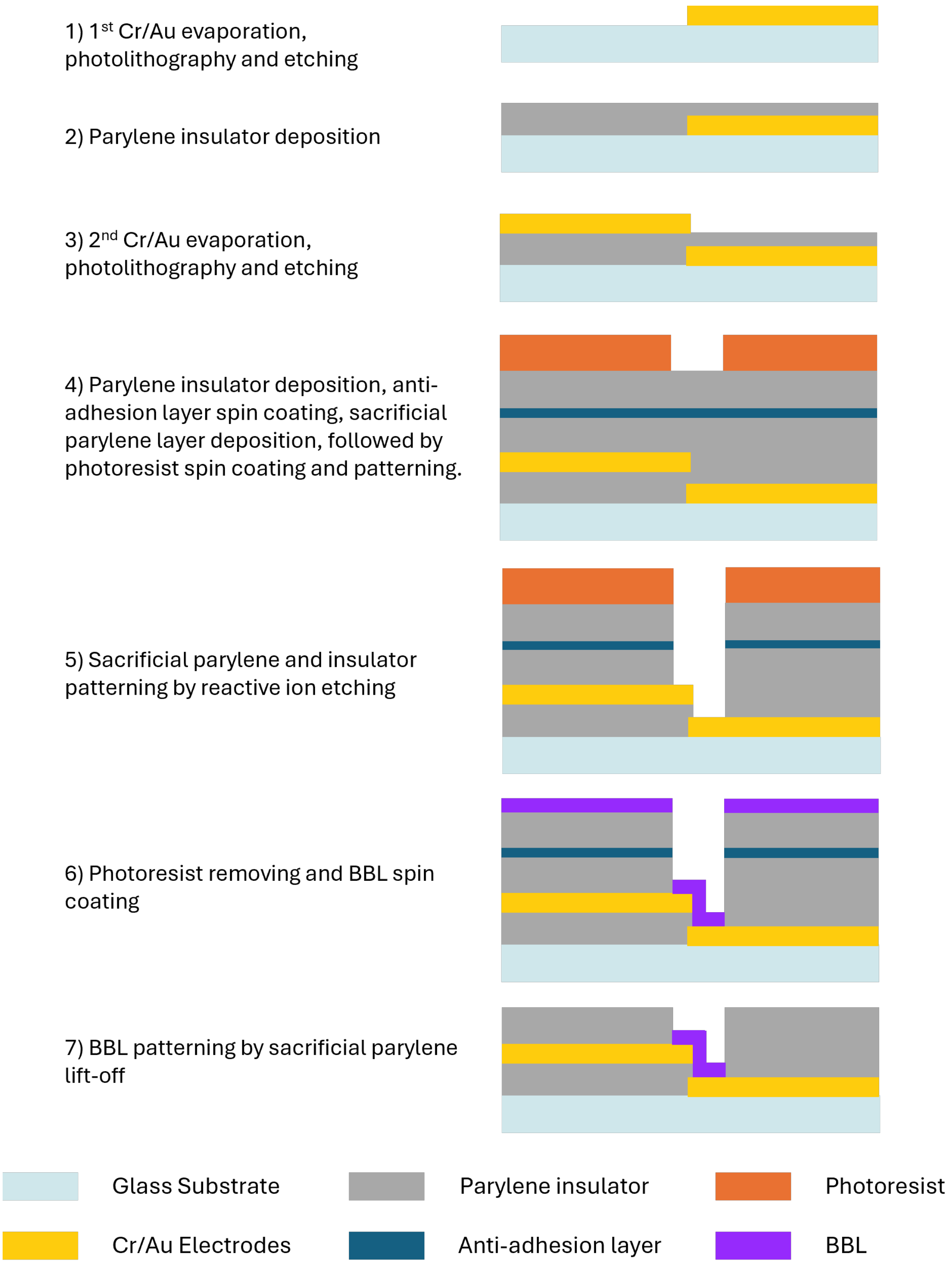

Supplementary Figure 25. Schematic of the fabrication process for a vertical 1T-OECN.

Reference

1. Harikesh, P. C. *et al.* Organic electrochemical neurons and synapses with ion mediated spiking. *Nat Commun* **13**, 901 (2022).
2. Harikesh, P. C. *et al.* Ion-tunable antiambipolarity in mixed ion–electron conducting polymers enables biorealistic organic electrochemical neurons. *Nat. Mater.* **22**, 242–248 (2023).
3. Harikesh, P. C., Tu, D. & Fabiano, S. Organic electrochemical neurons for neuromorphic perception. *Nat Electron* **7**, 525–536 (2024).
4. Yi, W. *et al.* Biological plausibility and stochasticity in scalable VO2 active memristor neurons. *Nat Commun* **9**, 4661 (2018).
5. Izhikevich, E. M. Which model to use for cortical spiking neurons? *IEEE Transactions on Neural Networks* **15**, 1063–1070 (2004).
6. Prescott, S. A. Excitability: Types I, II, and III. in *Encyclopedia of Computational Neuroscience* (eds. Jaeger, D. & Jung, R.) 1–7 (Springer, New York, NY, 2013). doi:10.1007/978-1-4614-7320-6_151-1.
7. Paugam-Moisy, H. & Bohte, S. Computing with Spiking Neuron Networks. in *Handbook of Natural Computing* (eds. Rozenberg, G., Bäck, T. & Kok, J. N.) 335–376 (Springer, Berlin, Heidelberg, 2012). doi:10.1007/978-3-540-92910-9_10.
8. Xie, R.-G., Chu, W.-G., Hu, S.-J. & Luo, C. Characterization of Different Types of Excitability in Large Somatosensory Neurons and Its Plastic Changes in Pathological Pain States. *International Journal of Molecular Sciences* **19**, 161 (2018).
9. Izhikevich, E. M. *Dynamical Systems in Neuroscience: The Geometry of Excitability and Bursting*. (MIT Press, 2010).

10. Somjen, G. G. *Ions in the Brain: Normal Function, Seizures, and Stroke*. (Oxford University Press, 2004).

11. Kim, U. & McCormick, D. A. The Functional Influence of Burst and Tonic Firing Mode on Synaptic Interactions in the Thalamus. *J. Neurosci.* **18**, 9500–9516 (1998).

12. Rotstein, H. G. Mixed-Mode Oscillations in Single Neurons. in *Encyclopedia of Computational Neuroscience* (eds. Jaeger, D. & Jung, R.) 1–9 (Springer, New York, NY, 2013). doi:10.1007/978-1-4614-7320-6_31-1.

13. Ganguly, C. *et al.* Spike frequency adaptation: bridging neural models and neuromorphic applications. *Commun Eng* **3**, 1–13 (2024).

14. Kumar, S., Williams, R. S. & Wang, Z. Third-order nanocircuit elements for neuromorphic engineering. *Nature* **585**, 518–523 (2020).

15. Softky, W. R. & Koch, C. The highly irregular firing of cortical cells is inconsistent with temporal integration of random EPSPs. *J. Neurosci.* **13**, 334–350 (1993).

16. Destexhe, A. & Contreras, D. Neuronal Computations with Stochastic Network States. *Science* **314**, 85–90 (2006).

17. Hodgkin, A. L. & Huxley, A. F. A quantitative description of membrane current and its application to conduction and excitation in nerve. *J Physiol* **117**, 500–544 (1952).

18. *Principles of Neural Science*. (McGraw Hill, New York, 2021).

19. Hutcheon, B. *et al.* Resonance, oscillation and the intrinsic frequency preferences of neurons. *Trends in Neurosciences* **23**, 216–222 (2000).

20. Destexhe, A. & Paré, D. Impact of Network Activity on the Integrative Properties of Neocortical Pyramidal Neurons In Vivo. *Journal of Neurophysiology* **81**, 1531–1547 (1999).

21. Economo, M. N., Noueihed, J., Martinez, J. J. & White, J. A. Neural Modeling. in *Neural Engineering* (ed. He, B.) 463–496 (Springer International Publishing, Cham, 2020). doi:10.1007/978-3-030-43395-6_16.
22. Lobov, S., Zhuravlev, M., Makarov, V. & Kazantsev, V. B. Noise Enhanced Signaling in STDP Driven Spiking-Neuron Network. *Mathematical Modelling of Natural Phenomena* **12**, 109–124 (2017).
23. Vardi, R., Tugendhaft, Y., Sardi, S. & Kanter, I. Significant anisotropic neuronal refractory period plasticity. *EPL* **134**, 60007 (2021).
24. Gidon, A. *et al.* Dendritic action potentials and computation in human layer 2/3 cortical neurons. *Science* **367**, 83–87 (2020).
25. Akbari, M., Hussein, S. M., Chou, T.-I. & Tang, K.-T. A 0.3-V Conductance-Based Silicon Neuron in 0.18 μm CMOS Process. *IEEE Transactions on Circuits and Systems II: Express Briefs* **68**, 3209–3213 (2021).
26. Binczak, S., Jacquir, S., Bilbault, J.-M., Kazantsev, V. B. & Nekorkin, V. I. Experimental study of electrical FitzHugh–Nagumo neurons with modified excitability. *Neural Networks* **19**, 684–693 (2006).
27. Stoliar, P., Schneegans, O. & Rozenberg, M. J. Biologically Relevant Dynamical Behaviors Realized in an Ultra-Compact Neuron Model. *Front. Neurosci.* **14**, (2020).
28. Ni, S. *et al.* Silicon Modeling of Spiking Neurons With Diverse Dynamic Behaviors. *IEEE Transactions on Computer-Aided Design of Integrated Circuits and Systems* **41**, 2199–2212 (2022).
29. Pickett, M. D., Medeiros-Ribeiro, G. & Williams, R. S. A scalable neuristor built with Mott memristors. *Nature Mater* **12**, 114–117 (2013).
30. Yuan, R. *et al.* A neuromorphic physiological signal processing system based on VO2 memristor for next-generation human-machine interface. *Nat Commun* **14**, 3695 (2023).

31. Beck, M. E. *et al.* Spiking neurons from tunable Gaussian heterojunction transistors. *Nat Commun* **11**, 1565 (2020).

32. Li, H. *et al.* Single-Transistor Neuron with Excitatory–Inhibitory Spatiotemporal Dynamics Applied for Neuronal Oscillations. *Advanced Materials* **34**, 2207371 (2022).

33. Li, H. *et al.* Boolean Computation in Single-Transistor Neuron. *Advanced Materials* **n/a**, 2409040.

34. Yao, Y. *et al.* An organic electrochemical neuron for a neuromorphic perception system. *Proceedings of the National Academy of Sciences* **122**, e2414879122 (2025).

35. Belleri, P. *et al.* Unravelling the operation of organic artificial neurons for neuromorphic bioelectronics. *Nat Commun* **15**, 5350 (2024).
